# Retinal Degenerations: Genetics, Mechanisms, and Therapies

**DOI:** 10.1155/2011/764873

**Published:** 2011-11-02

**Authors:** Ian M. MacDonald, Muna I. Naash, Radha Ayyagari

**Affiliations:** ^1^Department of Ophthalmology, University of Alberta, Royal Alexandra Hospital, 10240 Kingsway Avenue Room 2319, Edmonton, AB, Canada T5H 3V9; ^2^Department of Cell Biology, The University of Oklahoma Health Sciences Center, 940 Stanton L Young Boulevard, Oklahoma City, OK 73104, USA; ^3^Shiley Eye Center, University of California San Diego, 9415 Campus Point Drive, Room 206, La Jolla, CA 92093, USA

The cumulative results from genetic research and treatment protocols tested on cell and animal models have improved our understanding of pathobiology of inherited and degenerative eye diseases, and have lead to the development of a broad range of potential strategies to treat these conditions. In this special issue of the Journal of Ophthalmology, a series of articles provide a review of various areas of current eye research and clinical practice. 

A retinal degeneration phenotype is observed in several nonsyndromic and syndromic disorders, which are genetically and phenotypically heterogeneous. Usher syndrome, the commonest cause of deaf-blindness, is characterized by retinal degeneration, hearing loss, and, in some cases, abnormal vestibular function. Usher syndrome has been classified into three forms based on the age of onset, severity of retinal degeneration, and hearing loss. So far, nine genes associated with Usher syndrome have been identified. In this issue, Millán et al. “*An Update on the Genetics of Usher Syndrome*” review the genetics of Usher syndrome, providing a composite picture of the complexity of genetic and phenotypic heterogeneity as associated with retinal degenerations. 

As an introduction to basic mechanisms, Ozawa and colleagues “*Regulation of Posttranscriptional Modification as a Possible Therapeutic Approach for Retinal Neuroprotection*” discuss how the ubiquitin-proteosome system is recruited as part of chorioretinal inflammation and diabetic retinopathy. In patients with diabetic retinopathy, clinical electrophysiology will reveal an effect on the oscillatory potentials that can be explained by impaired synaptic function in retinal cells. We learn that two FDA-approved proteosome inhibitors, bortezomib and sorafenib, are now being tested in clinical trials, targeting the ubiquitin-proteosome system, and may have future applications in the treatment of retinal degenerations. More background on mechanisms is provided by Mitsura et al “*Effects of Calcium Ion, Calpains, and Calcium Channel Blockers on Retinitis Pigmentosa*” who discuss the central role that apoptosis plays in the pathways of retinal degeneration. Intracellular calcium increases with apoptosis, activating calpains that in turn trigger caspase-mediated events. They explain how calpain inhibitors and calcium channel antagonists may modulate photoreceptor degeneration and advocate for expanded human trials targeting this pathway. 

Several advances have been made in developing therapeutic strategies to treat retinal degenerations, and the success of these approaches to restore vision in patients with retinal degeneration may depend on the functional integrity of retinal ganglion cells. Using animal models, Margolis and Detwiler “*Cellular Origin of Spontaneous Ganglion Cell Spike Activity in Animal Models of Retinitis Pigmentosa*” demonstrate ganglion cell spike discharge occurs in the absence of photoreceptors and explain the cellular origin of this activity. Loss of photoreceptors in retinal degeneration can alter the synaptic activity of ganglion cells and understanding this phenomenon is critical for developing successful therapies for retinal degenerations. An article by Musarella and MacDonald “*Current Concepts in the Treatment of Retinitis Pigmentosa*” provides a synopsis of the current treatments of retinitis pigmentosa including the use of pharmacologic agents, nutritional therapies, stem cell approaches, artificial retinal implants, neuroprotective agents (CNTF, GDNF, and others), and gene therapy. Some of these approaches are exemplified in papers from this special issue, with more in depth discussion of the treatment of specific monogenic diseases such as Leber hereditary optic neuropathy (LHON) and animal models of human disease. Sullivan et al. “*Variables and Strategies in Development of Therapeutic PostTranscriptional Gene Silencing Agents*” present an overview on various strategies used in the development of therapies by post-transcriptional gene silencing. These strategies are effective in silencing the mutant target mRNA in dominant hereditary conditions or a normal wildtype mRNA that is over expressed. You and colleagues “*Efficient Transduction of Feline Neural Progenitor Cells for Delivery of Glial Cell Line-Derived Neurotrophic Factor Using a Feline Immunodeficiency Virus-Based Lentiviral Construct*” demonstrate how feline neural progenitor cells can be transduced with a lentiviral construct to deliver the neurotrophic factor, GDNF. These cells might then have a potential application, after intravitreal delivery, in the treatment of retinal degeneration. In the paper by Koilkonda and Guy “*Leber's Hereditary Optic Neuropathy-Gene Therapy From Benchtop to Bedside*, we understand the importance of cell and animal models to test new therapies for mitochondrial disease before introducing them into the clinical setting. The challenges of finding a treatment for a sudden onset, rare mitochondrial disorder, such as LHON, are illustrated by the unsuccessful effect in LHON patients of brimonidine, a conventional agent that has potential neuroprotective effects. Further, we see the wonder and potential of gene therapy for LHON as they introduce the concept of “allotropic” expression of a gene; essentially, introducing the normal gene, which is usually encoded as a mitochondrial gene, into the nucleus and then adding a sequence that would allow the protein to be targeted to its normal site of expression in the mitochondrion. Genes that are expressed in multiple cell types can be involved in causing degeneration in selected cell types. The article by Le “*Conditional Gene Targeting: Dissecting the Cellular Mechanisms of Retinal Degenerations*” describes conditional targeting of genes by a cre-lox-based approach which will enable targeting genes in specific cell types to understand the pathobiology of retinal degeneration observed in selected retinal layers.

Animal models offer an excellent opportunity to study disease pathology and evaluate therapeutic strategies. Among the various animal models, the mouse is a primary model of choice for retinal degeneration studies as the retinal morphology and physiology of these animals is well understood, their life span is shorter and studies on mice can be carried out in a cost-effective manner. In an article by Won et al. “*Mouse Model Resources for Vision Research*” 160 mutant mouse lines with ocular diseases including cataracts, retinal degeneration, and abnormal blood vessel formation have been described in detail. In addition to mouse models, other naturally occurring animal models also contributed significantly to our knowledge of retinal degenerations. In this issue, Narfström et al. “*The Domestic Cat as a Large Animal Model for Characterization of Disease and Therapeutic Intervention in Hereditary Retinal Blindness*” describe two feline models of human retinal dystrophies due to mutations in the *Cep290* and *Crx* genes. Cats with large eye size and a cone-rich, area centralis may serve as valuable models to study human retinal degeneration and evaluate therapeutic strategies. 

Recent advances in retinal degeneration research and the advent of new therapies improve our understanding of these conditions and provide hope for patients and families with retinal degenerations.

*Ian M. MacDonald* *Ian M. MacDonald* *Muna I. Naash* *Muna I. Naash* *Radha Ayyagari* *Radha Ayyagari*

